# Blockade of NR2A-Containing NMDA Receptors Induces Tau Phosphorylation in Rat Hippocampal Slices

**DOI:** 10.1155/2010/340168

**Published:** 2010-05-20

**Authors:** Julie Allyson, Eve Dontigny, Yves Auberson, Michel Cyr, Guy Massicotte

**Affiliations:** ^1^Département de chimie-biologie, Université du Québec à Trois-Rivières, Trois-Rivières, QC, Canada G9A 5H7; ^2^Novartis Institutes for BioMedical Research, 4002 Basel, Switzerland

## Abstract

Physiological activation of the N-methyl-D-aspartate (NMDA) subtype of glutamate receptors has been proposed to play a key role in both neuronal cell function and dysfunction. In the present study, we used selective NMDA receptor antagonists to investigate the involvement of NR2A and NR2B subunits in the modulatory effect of basal NMDA receptor activity on the phosphorylation of Tau proteins. We observed, in acute hippocampal slice preparations, that blockade of NR2A-containing NMDA receptors by the NR2A antagonist NVP-AAM077 provoked the hyperphosphorylation of a residue located in the proline-rich domain of Tau (i.e., Ser199). This effect seemed to be Ser199 specific as there was no increase in phosphorylation at Ser262 and Ser409 residues located in the microtubule-binding and C-terminal domains of Tau proteins, respectively. From a mechanistic perspective, our study revealed that blockade of NR2A-containing receptors influences Tau phosphorylation probably by increasing calcium influx into neurons, which seems to rely on accumulation of new NR1/NR2B receptors in neuronal membranes and could involve the cyclin-dependent kinase 5 pathway.

## 1. Introduction

The N-methyl-D-aspartate (NMDA) subtype of ionotropic glutamate receptors is known to play essential roles in the mammalian central nervous system [1–3]. For instance, in several pathological circumstances associated with neuronal damage, excessive levels of calcium influx through NMDA receptor channels are well recognized to promote cell death mechanisms, such as excitotoxicity and apoptosis [4, 5]. Over the years, however, a growing number of reports have revealed that, in contrast to the destructive effects of excessive NMDA receptor activity, synaptic NMDA receptor stimulation under physiological conditions could result in the activation of prosurvival mechanisms [6–9]. Along this line, tonic activation of NMDA receptors in hippocampal neurons was demonstrated to be important in maintaining synaptic stability, through a mechanism involving modulation of dendritic protein synthesis. In fact, it has been reported that tonic NMDA receptor activation acts as a crucial mechanism regulating calcium mobilization in neurons, as NMDA receptor deprivation rapidly increases the synaptic expression of surface GluR1 subunits and the incorporation of Ca^2+^-permeable AMPA receptors at synapses [10]. 

There are also several indications that physiological levels of NMDA receptor activation could play an active role in regulating cytoskeleton integrity and function. For example, a recent study by Fiumelli et al. [11] revealed that suppression of NMDA receptor activity by global antagonists (MK801 or AP5) can interfere with both phosphorylation and solubility of neurofilament subunit M in isolated cortical neurons. In this particular case, neurite outgrowth is promoted by the inactivation of NMDA receptors, suggesting that basal levels of NMDA receptor activity are crucial for regulating cytoskeleton stability and growth processes. Some authors have reported that tonic NMDA receptor activity in cerebellar granule cells and hippocampal neurons also regulates microtubule-associated protein 2 (MAP2) phosphorylation and neurite growth in the cerebellum [12, 13], while others have shown that activation of NMDA receptors in physiological conditions is likely to influence Tau phosphorylation in the hippocampal area [11, 14]. Tau proteins are well known for their involvement in the outgrowth of neural processes, the development of neuronal polarity, and the maintenance of normal neuron morphology [15]. Several investigations have demonstrated that disruption of normal Tau phosphorylation could be a key factor contributing to neurodegenerative disorders such as Alzheimer's disease (AD) [16–18].

Although the detailed molecular mechanisms by which NMDA receptors can regulate both physiological and pathophysiological processes remain to be elucidated, it has been proposed that NMDA receptors function may be highly dependent on the composition of their subunits, which are heteromeric assemblies of at least 1 NR1 subunit and various NR2 (A-D) subunits [19–21]. In the hippocampus, extensive evidence indicates that, in the mature stage, pyramidal cells mainly express NMDA receptors containing NR1/NR2A and NR1/NR2B subunits [22]. From a functional perspective, it has been argued by many that NR1/NR2A subunit activation could favour the action of prosurvival mechanisms, whereas NR1/NR2B subunit stimulation could lead to neuronal cell death by the involvement of various damaging signalling pathways [23, 24]. Accordingly, using different pharmacological agents, we observed that the tonic stimulation of NR2A-containing NMDA receptors in acute hippocampal slices might be a crucial component influencing Tau phosphorylation. 

## 2. Materials and Methods

### 2.1. Ethics Approval

Animal care procedures were reviewed by the Institutional Animal Care Committee of the Université du Québec à Trois-Rivières and found to be in compliance with guidelines of the Canadian Council on Animal Care.

### 2.2. Animals and Pharmacological Agents

Male Sprague-Dawley rats (6-7 weeks of age), purchased from Charles River Laboratories (Montréal, QC, Canada), were housed for 1 week prior to any experiments in a temperature-controlled room, with free access to laboratory chow and water. The selective NR2A antagonist NVP-AAM077 (NVP) was a gift from Dr. Yves Auberson (Novartis Pharma AG, Basel, Switzerland). NR2B (RO25-6981) and AMPA (NBQX) receptor antagonists were obtained from Tocris Bioscience (Ellisville, MO, USA), while the glycogen synthase kinase-3 beta (GSK-3*β*) inhibitor SB-216367 was procured from BioMol (Plymouth, PA, USA). Cyclin-dependent kinase 5 (roscovitine), calpain (calpeptin) as well as protease and phosphatase inhibitor cocktails were acquired from Calbiochem (San Diego, CA, USA). The membrane-impermeable and the membrane-permeable calcium chelator BAPTA were purchased from BioMol (Plymouth, PA, USA). The biotinylation reagent Sulfo-NHS-SS-Biotin was bought from Fisher Scientific (Nepean, ON, Canada). All other chemicals were supplied by Sigma-Alrich (Oakville, ON, Canada). 

### 2.3. Antibodies

Most antibodies reacting with Tau proteins were purchased from AbCam (Cambridge, MA, USA). The mouse polyclonal antibody Tau-5 was used (dilution 1 : 500) to estimate the total levels of Tau proteins in hippocampal extracts, along with rabbit polyclonal antibodies recognizing Tau phosphorylated at Ser199 (dilution 1 : 1,000), Ser262 (dilution 1 : 1,000), and Ser409 (dilution 1 : 1,000). GAPDH antibody also was purchased from AbCam, and rabbit polyclonal antibodies against NR1—(dilution 1 : 200), NR2A—(dilution 1 : 200), and NR2B-containing (dilution 1 : 200) NMDA receptors were obtained from Santa Cruz Biotechnology (San Diego, CA, USA). Rabbit anti-GluR1 (dilution 1 : 20) was provided by Calbiochem. Goat antirabbit or goat antimouse peroxydase-conjugated antibodies (dilution 1 : 5,000) and SuperSignal chemiluminescent substrate kits were from Pierce Chemical Co. (Rockford, IL, USA). 

### 2.4. Hippocampal Slices and Tissue Samples

Sprague-Dawley rats were anesthetized by isoflurane inhalation (Baxter Corp., Toronto, ON, Canada) and decapitated. Their brains were quickly removed and placed in cold cutting buffer containing 126 mM NaCl, 3.5 mM KCl, 1.2 mM NaH_2_PO_4_, 2.3 mM MgCl_2_, 1 mM CaCl_2_, 25 mM NaHCO_3_, and 11 mM glucose, saturated with 95%  O_2_/5% CO_2_ (pH 7.4). Coronal brain sections of 350 *μ*m containing the hippocampus were sliced in a Vibratome Series 1000 tissue sectioning system (Technical products international Inc., St. Louis, MO, USA). Sections were then transferred to artificial cerebrospinal fluid (ACSF) containing 126 mM NaCl, 3.5 mM KCl, 1.2 mM NaH_2_PO_4_, 1.3 mM MgCl_2_, 2 mM CaCl_2_, 25 mM NaHCO_3_, and 11 mM glucose, bubbled continuously with 95% O_2_/5%  CO_2_ at 32°C. The brain sections were preincubated for 60 minutes before pharmacological treatment. After pharmacological treatment, hippocampal slices were dissected from the brain sections and homogenized in ice-cold RIPA lysis buffer containing 50 mM Tris-HCl, 150 mM NaCl, 1% Triton X-100, 0.25% sodium deoxycholate, and 1 mM EDTA supplemented with protease and phosphatase inhibitor cocktails.

### 2.5. Cell Surface Biotinylation

Hippocampal slices were incubated for 2 hours with or without NVP-AAM0077. After several washes with ACSF bubbled constantly with 95% O_2_/5%  CO_2_, each hippocampal slice was incubated in 1 mg/ml sulfosuccinimidyl-2-(biotinamido) ethyl-dithiopropionate (sulfo-NHS-SS-biotin), followed by several washes with sulfo-NHS-SS-biotin blocking reagent (50 mM NH_4_Cl in PBS containing 1 mM MgCl_2_ and 0.1 mM CaCl_2_) at 4°C to quench free sulfo-NHS-SS-biotin, followed by more than a few washes in ACSF at 4°C. Each slice was homogenized in 100 *μ*l of Tris acetate buffer (50 mM, pH 7.4) containing 1 mM EGTA, 1 mM EDTA, and numerous protease and phosphatase inhibitors (leupeptin 10 *μ*M, phenylmethylsulfonyl fluoride 1 *μ*g/ml, and N-tosyl-L-phenylalanine chloromethyl ketone 1 *μ*g/ml). The samples were centrifuged for 10 minutes at 11,070 rpm at 4°C. The supernatants were removed and the pellets were suspended in fresh, ice-cold Tris acetate buffer. Streptavidin beads (50 *μ*l/300 *μ*g of proteins) were washed 3 times with Tris acetate buffer. Biotinylated samples (300 *μ*g of protein) were added to the beads and mixed at room temperature for 4 hours. The beads were recovered by brief centrifugation, and the supernatants were removed. The beads were washed 3 times with Tris acetate buffer, and biotinylated samples were eluted from the beads with 4 X sodium dodecyl sulphate-10% polyacrylamide gel electrophoresis (SDS-PAGE) loading buffer (containing *β*-mercapto-ethanol) at 100°C for 10 minutes. The supernatants were removed, and biotinylated protein levels were detected by SDS-PAGE and immunoblotting.

### 2.6. Western Blotting

Protein levels extracted from rat hippocampus sections were measured by Bradford assay (Bio-Rad, Hercules, CA, USA). Electrophoresis of protein lysates (40 *μ*g), except for the NR2B subunits which required 80 *μ*g of homogenized protein, was performed on 10% polyacrylamide gel (SDS-PAGE). Separated proteins were transferred onto nitrocellulose membranes and nonspecific binding sites were blocked by incubation for 1 hour at room temperature in phosphate-buffered saline, pH 7.4, containing 5% bovine serum albumin (BSA fraction V) purchased from Fisher Scientific (Pittsburgh, PA, USA). Then, selected primary antibodies were incubated overnight at 4°C. After several washes with 0.1% Tween 20, the blots were incubated for 2 hours at room temperature in specific secondary HRP-conjugated antibody solution. Both primary and secondary antibodies were diluted in TBS/0.1% Tween 20/1% BSA. Immunoreactivity was visualized by chemiluminescence reactions, and the intensity of the bands was quantified by densitometric scanning through Vision Work LS software (UVP Bioimaging, Upland, CA, USA). The densitometry data were expressed as relative optical density.

### 2.7. Statistical Analysis

The results are expressed as mean average ± SEM. Statistical significance of the changes was determined using Graph Prism version 5.0 (Graph Pad Software, San Diego, CA, USA). *P* < .05 values were considered as statistically significant. 

## 3. Results

### 3.1. Blockade of NR2A-Containing NMDA Receptors Selectively Enhances Phosphorylation of Tau Proteins at Ser199 Residues

**Figure 1:**
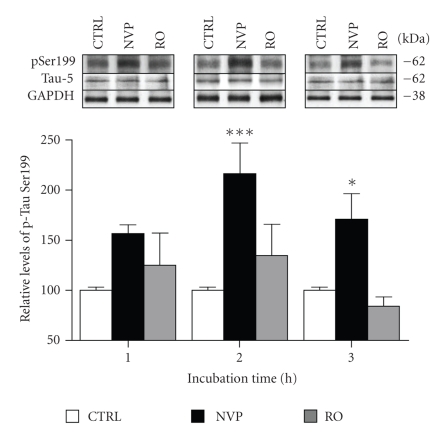
Blockade of NR2A-containing NMDA receptors induces Tau phosphorylation at Ser199 site in rat hippocampal slices. Phosphorylation and protein levels were estimated by Western blotting on cell extracts (40 *μ*g proteins) obtained from acute hippocampal slices treated with 2 NMDA receptor antagonists for periods ranging from 1 to 3 hours. Phosphorylated Tau levels at Ser199, expressed relative to total Tau (Tau-5) levels, were measured in slices treated with 50 nM NVP and 1 *μ*M RO. The data were expressed as percentage of control values and are means ± SEM of 3 measurements per cell extract obtained from 7 different rats. Statistical analysis using two-way ANOVA followed by the *post hoc* Bonferroni test revealed that there was a main effect between treatment (F(2,54) = 16.370, *P* < .0001), no effect between time (F(2,54) = 2.509, *P* = .091) and no significant interaction between treatment and time (F(4,54) = 1.337, *P* = .268). *P < .05, ***P < .001, drug-treated versus control.

In this study, we investigated the influence of tonic NMDA receptor activity on Tau status by quantifying phosphorylation and protein levels in the hippocampus. Acute hippocampal slices from rats were treated for different time periods with NMDA receptor antagonists and then processed by Western blotting. We first examined Tau phosphorylation levels on Ser199 after preincubating hippocampal slices with NVP-AAM077 (NVP) and R025-6981 (RO), 2 compounds that preferentially block, respectively, NR2A- and NR2B-containing NMDA receptors. Our experiments were performed with 50 nM NVP and 1 *μ*M RO, concentrations that are known to be highly selective for NR2A and NR2B, respectively [25, 26]. In initial experiments, we observed that hippocampal tissues were strongly and consistently stained with an antibody recognizing the phosphorylated Ser199 epitope of a Tau isoform estimated to 62 kDa (Figure 1, top panels). As presented in the Figure 1 histogram, we observed that this Tau isoform became progressively hyperphosphorylated at the Ser199 residue after blockade of NR2A-containing NMDA receptors with NVP (Figure 1, black bars). In fact, when normalized with Tau-5 (an antibody that recognized phosphate-independent epitopes of Tau), it became evident that overtime NVP elevated phosphorylated Tau levels at its Ser199 site, with a maximal increase observed in slices preincubated for a period of 2 hours (*n* = 5, *P* < .01). Time-course analysis showed, however, that Ser199 was not subjected to substantial change in phosphorylation after exposure to the NR2B antagonist RO (Figure 1, grey bars). It is noteworthy that treatments of rat hippocampal slices with both NVP and RO failed to produce significant changes in Tau-5 staining intensity (Figure 1, top panels), indicating that Ser199 hyperphosphorylation resulting from blockade of NR2A-containing receptors is not dependent on variations in Tau synthesis and/or degradation.

**Figure 2:**
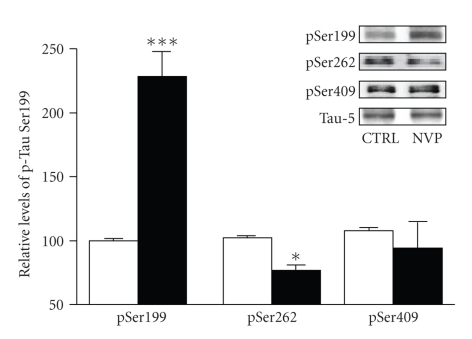
Blockade of NR2A-containing NMDA receptors is not associated with increased Tau phosphorylation levels at Ser409 and Ser262 sites. Phosphorylation and protein levels were estimated by Western blotting on cell extracts (40 *μ*g proteins) obtained from acute hippocampal slices treated with 50 nM NVP for 2 hours. Phosphorylated Tau levels, expressed relative to total Tau (i.e., Tau-5) levels, were measured using antibodies raised against Tau phosphorylated at Ser199, Ser262, and Ser409. The data were expressed as percentage of control values and are means ± SEM of 3 measurements per cell extract obtained from 6 different rats. Since these experiments were independently performed, we determined statistical significance using unpaired *T*-Test. *P < .05, ***P < .0001, NVP-treated versus control.

So far, Tau has been found to possess 70 different phosphorylation sites. The Ser199 epitope is known to be located in the proline-rich domain of Tau proteins. This observation led us to investigate whether other Tau phosphorylation sites are also under the influence of NMDA receptors containing NR1/NR2A subunits. Figure 2 shows that preincubation of hippocampal slices with NVP (50 nM for 2 hours) failed to elicit any changes in phosphorylation at the Ser409 residue of Tau proteins, a phosphorylation site positioned in the C-terminal domain. Similarly, Western blotting experiments indicated that phosphorylation of an epitope located in the microtubule-binding domain of Tau (Ser262) was not accentuated after blockade of NR2A-containing NMDA receptors. If anything, quantification and averaging of data obtained from several slices indicated that, in contrast to Ser199, after 2-hour NVP exposure, phosphorylated Ser262 levels were slightly but significantly reduced (Figure 2). 

### 3.2. Role of Calcium and Cdk5 Signalling in NVP-Induced Tau Phosphorylation

**Figure 3:**
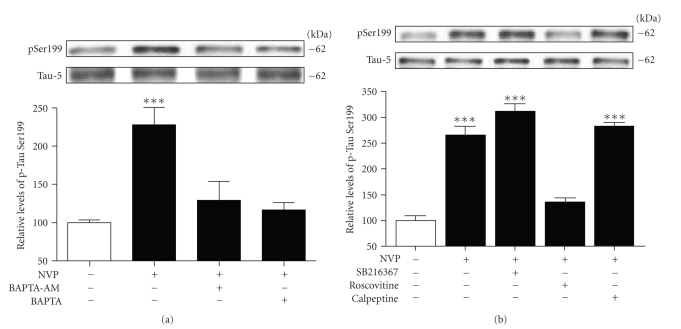
NVP-induced Tau phosphorylation is mediated by calcium and the Cdk5 pathway. (a) Phosphorylated Tau levels at Ser199 were estimated by Western blotting on cell extracts obtained from acute hippocampal slices treated with 50 nM NVP for 2 hours alone or in combination with 10 *μ*M BAPTA-AM or 10 *μ*M BAPTA. The data are expressed relative to total Tau (i.e., Tau-5) levels. (b) As in A, except for the GSK-3*β* inhibitor SB216367 (10 *μ*M), the Cdk5 inhibitor roscovitine (10 *μ*M), or the calpain inhibitor calpeptine (10 *μ*M) were employed. The data were expressed as percentage of control values and are means ± SEM of 3 measurements per cell extract obtained from 5 different rats. Statistical analysis was performed by one-way ANOVA followed by Neuman-Keuls' post hoc test. ***P < .001, drug-treated versus control.

Previous studies in rat hippocampal cultures indicated that NMDA receptor antagonists, such as MK801, rapidly increased calcium permeability in neurons [10]. Thus, we sought to investigate whether NVP-induced phosphorylation might, in fact, be dependent on calcium mobilization. We observed that preexposure of hippocampal slices to BAPTA-AM, a cell-permeable agent with very high affinity for calcium, completely abolished the increased levels of phosphorylated Tau at its Ser199 residue (Figure 3(a)). Similarly, preexposure of hippocampal slices to the cell-impermeable form of BAPTA also completely blocked the NVP-induced phosphorylation of Ser199 (Figure 3(a)), indicating that Tau hyperphosphorylation after inhibition of NR2A-containing receptors mainly relies on calcium entrance from the extracellular space. 

The observation that the effect of NVP is dependent on calcium entrance predicts that selective phosphorylation at Ser199 residue could involve the action of protein kinases. The proline-directed protein kinases known to influence phosphorylation of Ser199 residue include GSK-3*β* and Cdk5 (Figure 3(b)). Therefore, based on this information, we examined whether inhibitors of Cdk5 or GSK-3*β* signalling pathways might prevent NVP-induced phosphorylation at the Ser199 site. To assess a possible role of GSK-3*β* in Tau phosphorylation induced by blockade of NR2A-contaning receptors, we used the selective GSK-3*β* inhibitor SB216367. In these experiments, the inhibitor was applied 45 minutes before NVP exposure to ensure optimal inhibition of GSK-3*β*. We observed that preexposure of slices to 10 *μ*M of SB216367 did not interfere with NVP-induced phosphorylation. On the contrary, Ser199 hyperphosphorylation in slices preexposed to 10 *μ*M roscovitine was totally prevented; indicating that NVP-induced Tau phosphorylation at Ser199 primarily involves the Cdk5 pathway. We also evaluated whether calpain-mediated activation of Cdk5 could be responsible for the effects on Tau phosphorylation. Here, calpeptin did not prevent NVP-induced Tau phosphorylation, suggesting that this phenomenon occurs independently of calpain activation (Figure 3(b)).

### 3.3. NVP-Induced Tau Phosphorylation Relies on Activation of NR2B-Containing NMDA Receptors

**Figure 4:**
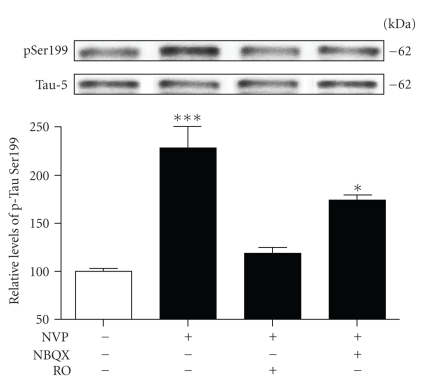
NR2B-containing receptors play a role in Tau phosphorylation induced by NVP. Phosphorylated Tau levels at Ser199 were estimated by Western blotting on cell extracts obtained from acute hippocampal slices treated with 50 nM NVP for 2 hours alone or in combination with 10 *μ*M NBQX and 10 *μ*M RO25-6981. The data, expressed relative to total Tau (i.e., Tau-5) levels, are means ± SEM of 3 measurements per cell extract obtained from 4 different rats. Statistical analysis was performed by one-way ANOVA followed by Neuman-Keuls' post hoc test. *P < .05, ***P < .001, drug-treated versus control.

**Figure 5:**
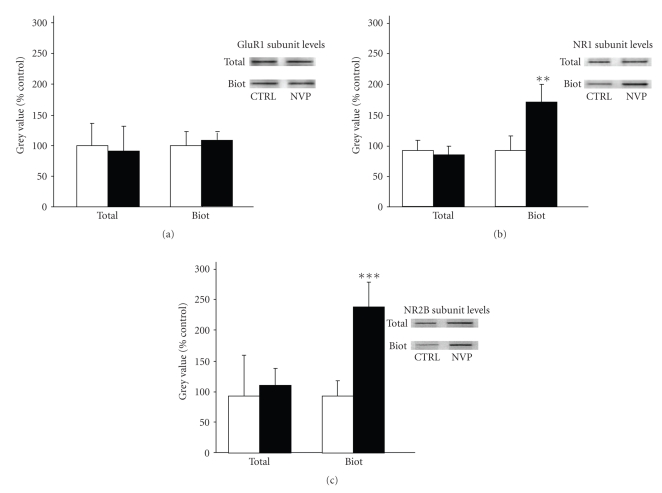
Blockade of NR2A-containing NMDA receptors is associated with increased levels of NR1 and NR2B subunits in biotinylated membranes. Glutamate receptor subunit levels were estimated by Western blotting on biotinylated membranes obtained from acute hippocampal slices treated with 50 nM NVP for 2 hours. Biotinylated NR1, NR2B and GluR1 subunit levels were normalized with respective total protein levels estimated in homogenates of hippocampal slices incubated with or without NVP. The data are means ± SEM of 3 measurements obtained from 4 different rats. Since these experiments were independently performed, we determined statistical significance using unpaired *T*-Test. **P < .01, ***P < .001, NVP-treated versus control.

Taken together, the above findings indicate that blockade of NR2A-containing NMDA receptors promotes Tau phosphorylation at Ser199 residue, implicating both calcium and the Cdk5 signalling pathway. The exact mechanisms by which NVP induces calcium mobilization, however, remain to be clarified. One possibility is that blockade of NR2A-containing NMDA receptors could have led to calcium entrance in neurons by favouring the dysregulation of other glutamate receptor subtypes. Thus, we initiated a series of experiments to determine the effects of glutamate receptor antagonisms on NVP-induced Tau phosphorylation in rat hippocampal slices. Here, we report the results obtained with an antagonist acting on the AMPA subtype of glutamate receptors (NBQX) and the antagonist acting on NR2B-containing NMDA receptors (i.e., RO). Figure 4 shows that preexposure of hippocampal slices to 10 *μ*M NBQX did not significantly reduce Tau phosphorylation at Ser199 residue resulting from inhibition of NR2A-containing receptors by NVP. However, NVP-induced Tau phosphorylation was completely reversed by the preincubation of slices in the presence of RO, suggesting that the ability of NVP to enhance phosphorylation of the Ser199 epitope is possibly dependent on alterations of NR2B-containing NMDA receptors. Indeed, we decided to test this scenario by measuring the surface expression of both AMPA and NMDA receptor subunits on biotinylated membranes [27]. As shown in Figure 5(a), the surface level of GluR1 subunits of AMPA receptors was not significantly modified in slices treated with NVP. In contrast, a significant effect on NR1 subunits of NMDA receptors was observed in hippocampal slices 2 hours after NVP exposure. The NR2A antagonist was found to increase NR1 subunit levels by more than 60% in biotinylated membranes prepared from hippocampal slices (Figure 5(b)), while similar results were obtained with NR2B subunit levels (Figure 5(c)).

## 4. Discussion

**Figure 6:**
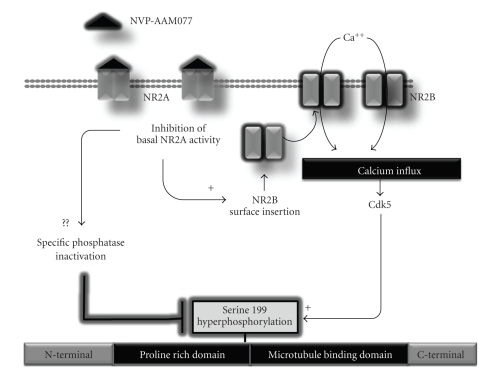
Working model of NVP-induced Tau phosphorylation. Blockade of NR2A-containing receptors appears to initiate the insertion of new functional NMDA receptors containing a high proportion of NR2B subunits in neuronal plasma membranes, promoting calcium accumulation. Through an unknown mechanism, Cdk5 activity would selectively enhance the phosphorylation of Ser199 residue in the proline-rich domain of Tau. In parallel, blockade of NR2A-containing receptors may reduce specific phosphatase activity which could have an impact on Tau phosphorylation at Ser199.

In this study, we examined the effects of inhibition of NR2A- and NR2B-containing NMDA receptors on Tau phosphorylation in acute hippocampal slices. We demonstrated that pharmacological blockade of NR2A-containing NMDA receptors induces a robust and selective increase of the phosphorylation level of the serine residue Ser199 of Tau. Moreover, we showed that calcium mobilization and activation of the Cdk5 signalling pathway are directly involved in this effect, which probably rely on the insertion of new NR1/NR2B subunits in neuronal membranes. A putative biochemical model that accounts for the control of Tau phosphorylation by NVP is illustrated in Figure 6.

According to our results, inactivation of NR2A-containing NMDA receptors by NVP in acute hippocampal slices elicits a significant increase in the phosphorylation state of Tau, suggesting that the tonic activity of these receptors contributes to limit Tau phosphorylation in basal physiological conditions. This is in line with previous in vitro studies showing that suppression of NMDA receptor activity by global antagonists (MK801 or AP5) can enhance Tau phosphorylation [11]. In the present report, we demonstrate that hyperphosphorylation of Ser199 residue resulting from inactivation of NR2A-containing NMDA receptors is totally abrogated by the nonpermeable form of BAPTA, indicating that the effect likely relies on calcium entry into neuronal cells. In this context, we have investigated the potential signalling pathways underlying NVP-induced tau phosphorylation in hippocampal slice preparations. The phosphorylation state of Tau epitopes is known to be under the regulation of various kinase pathways, which are directly or indirectly influenced by calcium ions. Generally, Tau is phosphorylated by 2 major categories of kinases, which are divided according to motif specificity: proline-directed protein kinases (PDPK) and nonproline-directed protein kinases (non-PDPK) [28]. Cdk5, mitogen-activated protein kinase, and several stress-activated protein kinases are included in the PDPK family. GSK-3*β* is habitually described as a PDPK, although proline is not always necessary for Tau phosphorylation by GSK-3*β*. Non-PDPK include cyclic AMP-dependent protein kinase A, calcium- and calmodulin-dependent protein kinase II, and microtubule affinity regulating kinase (MARK), the mammalian homologue of PAR-1 present in *Drosophila * [29]. MARK selectively phosphorylates a KXGS motif, within the microtubule binding repeat domains of Tau (serine residues at 262, 293, 324, and 356) [28]. The present data show that blockade of NR2A subunits with NVP significantly increased the phosphorylation of Ser199 epitope located in the proline-rich domain of Tau. From a mechanistic perspective, we demonstrated that this effect is possibly not dependent on GSK-3*β* activity, since blockade of the system by SB216367 had no effect on NVP-induced phosphorylation at Ser199 residue. However, application of a specific inhibitor of the Cdk5 pathway completely abolished NVP-induced Tau phosphorylation at Ser199 residue. 

Indeed, we have yet to fully characterize the mechanism by which Cdk5 enhances Tau phosphorylation at Ser199 but, according to the present investigation, this phenomenon appears to be independent of the activation of calpain enzymes, which are known to favour intracellular accumulation of the potent p25 activator of Cdk5 [30, 31]. In this line, our findings imply that other Cdk5 activators might be involved in the above-mentioned effects of NVP on Tau. This hypothesis is strongly supported by recent studies showing that Cdk5 activation can depend on IC53 production, a new potential activator of this kinase system [32]. In terms of cellular distribution, there are several indications that Tau proteins mainly localised in axonal compartments of neurons. Predictably, because of the preferential localization of NR2A-containing receptors in synapses, our findings implicate potential biochemical links between a presumable increase in dendritic calcium and subsequent stimulation of axonal Cdk5 in neurons subjected to NMDA receptor deprivation. However, as Tau proteins are also known to be localized in somatodendritic compartments of neurons, our results highlighted the need to also explore the possibility that NR2A-containing NMDA receptors could differentially influence Tau phosphorylation in axonal and somatodendritic compartments of neurons.

There are several lines of evidence that phosphorylation of other cytoskeletal proteins is accentuated after inhibition of NMDA receptors [11, 13]. For instance, it has been proposed that tonic NMDA receptor activation plays an essential role in limiting MAP2 phosphorylation in hippocampal slices through a mechanism involving stimulation of the calcium/calmodulin-dependent protein phosphatase calcineurin [13, 14]. Accordingly, one can speculate that the effect reported here would be dependent on inhibition of phosphatase activities after the blockade of NR2A-containing NMDA receptors by NVP. However, although this antagonist was found to markedly enhance the phosphorylation of Ser199 residue, it did not similarly augment the phosphorylation of other residues located in both the C-terminal (Ser409) and microtubule-binding (Ser262) domains of Tau, suggesting that dephosphorylation processes are not impaired by the blockade of NR2A-containing receptors. Nevertheless, it is worth mentioning that according to previous studies, Ser199 residues might represent unusual phosphorylation sites of Tau as they appear to be influenced by a very specific type of phosphatase activity (i.e., PP5) [33]. Indeed, experiments are required to directly examine whether inhibition of NR2A-containing receptors could lead to preferential reduction of PP5 activity in hippocampal slices. Independently of the mechanisms involved, the current study reinforces the recent observation that the serine residues of Tau can be differentially modulated in diverse circumstances. Along this line, by interfering with PLA2 activity in embryonic rat hippocampal neurons, De-Paula et al. [34] observed that Tau proteins may become hyperphosphorylated on Ser214 residue, sparing other epitopes, including Ser199, Ser202, Ser205, and Ser396 phosphorylation sites. 

The biochemical demonstration that NVP-induced Tau phosphorylation is completely abolished by preexposure of slices to RO indeed supports the notion that NMDA receptor inhibition influences the molecular properties of this neurotransmission system. Our results are in line with several reports showing that the number of NMDA receptors, the composition of their subunits and their postsynaptic linkers can be altered after the administration of NMDA receptor antagonists, including MK-801, ethanol, and phencyclidine [24, 35–37]. It is of interest that according to the present investigation, a significant increase in the levels of NR1 and NR2B subunits was observed after the blockade of NR2A-containing receptors, suggesting that the molecular mechanism underlying NVP-induced Tau phosphorylation in acute hippocampal slices might involve NMDA receptor enrichment at the membrane surface. We do not know the mechanism through which inhibition of NR2A-containing NMDA receptors could up-regulate NR1/NR2B subunits on hippocampal membranes. It is worth mentioning, however, that the surface expression and mobility of NR2A- and NR2B-containing receptors are differentially regulated, depending on scaffolding proteins interacting with the NR2 subunits. For instance, surface NR2B-containing NMDA receptors appear to be more mobile within neurons, possibly due in part to preferential interaction with synapse-associated protein 102 (SAP-102), over postsynaptic protein 95 [38]. Indeed, it remains to be determined whether NR1/NR2B receptor enrichment at the membrane surface depends on higher expression of NR2B subunit-SAP-102 complexes after NVP application. It is interesting that calcium mobilization is one of the consequences of NMDA receptor inactivation by global antagonists through mechanisms involving the incorporation of calcium-permeable AMPA receptors in neuronal membranes [10]. This issue needs to be further explored, but the present investigation strongly indicates that this scenario does not account for the capacity of NVP to induce Tau phosphorylation.

### 4.1. Summary and Conclusions

The current study argues that tonic activation of NR2A-containing NMDA receptors is required to limit Tau hyperphosphorylation at its Ser199 site. It is indeed premature to speculate on the functional significance of this effect, and the next challenge resulting from our observation will be to directly demonstrate that such increases in Tau phosphorylation may engage alterations in hippocampal functions. As reported previously, Tau predominantly localizes to neuronal axons where it modulates the stability and assembly of microtubules [39, 40]. In so doing, Tau generates a partially stable, but still dynamic, state in microtubules that is important for axonal growth and effective axonal transport [41]. In addition to binding microtubules, some but not all studies provide evidence that Tau can interact, either directly or indirectly, with actin and affect actin polymerization as well as the interaction of actin filaments with microtubules [42, 43]. Furthermore, Tau appears to interact with the plasma membrane and with several proteins involved in signal transduction [44–52]. From a pathological perspective, Tau dysfunction resulting from biochemical defects (i.e., aberrant phosphorylation, truncation, and glycosylation) has been proposed to be an important factor contributing to the initiation and development of several neuropathological conditions such as AD [16, 28, 53–56]. Thus, the fact that NR2A subunits are down regulated [57], coupled with the observation that hyperphosphorylation of Tau at Ser199 is present in the early stage of this disease [54], strongly suggests that the effect reported here may have interesting implications for understanding the mechanisms of AD. Collectively, our findings suggest that drugs acting as NMDA receptor antagonists could increase Ca^2+^ influx through inhibition of NR2A-containing receptors and enrichment of NR1/NR2B subunits. Such a change in receptor subunit composition could theoretically favour the appearance of adverse neuropathological effects [58], and should be evaluated further.
